# Identifying and Validating Clusters of Menopausal Symptoms Across Reproductive Stages

**DOI:** 10.1093/geroni/igaf122.3959

**Published:** 2025-12-31

**Authors:** Alan Rathbun, Rebecca Brotman, Joanna Borgogna, Sarah Brown, Jacques Ravel, Michelle Shardell

**Affiliations:** University of Maryland School of Medicine, Baltimore, Maryland, United States; University of Maryland School of Medicine, Baltimore, Maryland, United States; McLaughlin Research Institute, Great Falls, Montana, United States; University of Maryland School of Medicine, Baltimore, Maryland, United States; University of Maryland School of Medicine, Baltimore, Maryland, United States; University of Maryland School of Medicine, Baltimore, Maryland, United States

## Abstract

Biological changes during the menopausal transition produce symptoms across multiple domains, yet clinical profiles remain poorly understood, hindering screening and targeted care. This study aimed to identify and validate menopausal symptom clusters and examine their variation across reproductive stages. Women aged 35–60 years (n = 741) enrolled in the Human Papillomavirus in Perimenopause study were followed for two years and completed four semiannual follow-up visits assessing genitourinary, vasomotor, other physical, and mood-related menopausal symptoms. Latent class models were fit using all available data (2,360 person-visits) to identify symptom clusters. Models with two to six classes were compared via fit statistics, posterior probabilities, item-response probabilities, and interpretability. Participants were assigned to the symptom cluster for which they had the highest posterior probability. Four classes emerged: the multi-domain cluster (21.0%) had high probabilities for all symptoms; the asymptomatic cluster (30.6%) had uniformly low symptom burden; the mood/somatic cluster (27.0%) showed elevated psychological and non-specific physical symptoms; and the genitourinary/vasomotor cluster (21.4%) was marked by vaginal dryness, decreased sexual interest, and vasomotor symptoms. Age and the proportion of postmenopausal women were significantly higher in the multi-domain (49.8 years; 47.1%) and genitourinary/vasomotor (50.8 years; 46.3%) clusters than in the mood/somatic and asymptomatic clusters. These participants were also more likely to exhibit clinical signs of genitourinary syndrome of menopause, have lower current hormone use and estradiol levels, and were less likely to have Lactobacillus-dominant vaginal microbial communities. Menopausal symptoms cluster differently across reproductive stages, which may inform screening and targeted care.

